# Substance abuse behaviors among university freshmen in Iran: a latent class analysis

**DOI:** 10.4178/epih.e2018030

**Published:** 2018-07-02

**Authors:** Kourosh Kabir, Ali Bahari, Mohammad Hajizadeh, Hamid Allahverdipour, Mohammad Javad Tarrahi, Ali Fakhari, Hossein Ansari, Asghar Mohammadpoorasl

**Affiliations:** 1 School of Medicine, Alborz University of Medical Sciences, Karaj, Iran; 2 Department of Statistics and Epidemiology, Faculty of Health, Tabriz University of Medical Sciences, Tabriz, Iran; 3 School of Health Administration, Faculty of Health, Dalhousie University, Halifax, Canada; 4 Research Center of Psychiatry and Behavioral Sciences, Tabriz University of Medical Sciences, Tabriz, Iran; 5 Department of Epidemiology and Biostatistics, School of Public Health, Esfahan University of Medical Sciences, Esfahan, Iran; 6 Health Promotion Research Center, Zahedan University of Medical Sciences, Zahedan, Iran

**Keywords:** Substance abuse, Risk-taking behaviors, University freshman students, Water-pipe smoking, Tobacco smoking, Iran

## Abstract

**OBJECTIVES:**

Substance abuse behaviors among university freshmen in Iran are poorly understood. This study aimed to identify, for the first time, subgroups of university freshmen in Iran on the basis of substance abuse behaviors. Moreover, it examined the effects of socio-demographic characteristics on membership in each specific subgroup.

**METHODS:**

Data for the study were collected cross-sectionally in December 2013 and January 2014 from 4 major cities in Iran: Tabriz, Qazvin, Karaj, and Khoramabad. A total of 5,252 first-semester freshmen were randomly selected using a proportional cluster sampling methodology. A survey questionnaire was used to collect data. Latent class analysis (LCA) was performed to identify subgroups of students on the basis of substance abuse behaviors and to examine the effects of students’ socio-demographic characteristics on membership in each specific subgroup.

**RESULTS:**

The LCA procedure identified 3 latent classes: the healthy group; the hookah experimenter group; and the unhealthy group. Approximately 82.8, 16.1, and 2.1% of students were classified into the healthy, hookah experimenter, and unhealthy groups, respectively. Older age, being male, and having a family member or a close friend who smoked increased the risk of membership in classes 2 and 3, compared to class 1.

**CONCLUSIONS:**

Approximately 2.1% of freshmen exhibited unhealthy substance abuse behaviors. In addition, we found that older age, being male, and having a close friend or family member who smoked may serve as risk factors for substance abuse behaviors.

## INTRODUCTION

Adolescence and youth are unique stages of life and growth that offer opportunities and challenges for health promotion [1]. Adolescents often undergo numerous changes in terms of major individual characteristics, as well as changes that are the starting point of several behaviors in adulthood, such as drug abuse, sexual activities [2], and other high-risk behaviors [3]. Thus, these stages provide exceptional opportunities for helping individuals to establish and adopt healthy behavioral patterns to promote their present and future health [4].

Substance abuse behaviors (smoking, alcohol, drugs, marijuana, and other prohibited drugs) can have negative impacts on the well-being and overall development of adolescents and youths [5]. Considering these behaviors during adolescence and youth is particularly important because they are associated with several causes of death and illness during these periods [6]. Moreover, due to the specific characteristics of youth, high-risk behaviors in this stage can have long-lasting negative effects on the development and over-all health of society. Thus, youth is considered to be a uniquely important period for investigating high-risk behaviors [7].

Drug abuse is a major public health problem among adolescents globally [8]. Tobacco leads to the deaths of 7 million people annually, and nearly 80% of smokers live in low- and middle-income countries [9]. Moreover, smoking hookah causes irreparable damage to various body organs [10].

Studies among university students around the world have reported prevalence rates of 8.6 to 28.6% [9,11,12] and 14.3 to 34.0% for cigarette and hookah smoking in the past 30 days, respectively [13-17]. A 2012 study of Iranian university students reported prevalence rates of 15.8, 8.5, 8.0, and 7.6% for cigarette smoking, hookah smoking, alcohol use in the past month, and drug abuse, respectively [18]. Strong relationships exist between hookah, cigarette, and alcohol use among students [19]. Recent studies have also shown that high-risk behaviors usually co-occur (e.g., the cooccurrence of alcohol abuse and violence [20], alcohol abuse and high-risk sexual behaviors [21], alcohol abuse and smoking [22], and drug abuse and high-risk sexual behaviors [23]).

Entering college is associated with a sense of independence and separation from family among young adults, as college provides a new social environment where alcohol and drugs are commonly used for entertainment [24]. A study among freshmen college students in the US showed that hookah use in the past 30 days increased in the first academic year, and the highest prevalence of hookah use onset was in the first 2 months of academic instruction [25]. Freshmen are especially vulnerable to drug abuse [25], and the onset of drug abuse among students is typically in the first year of college [26], although the following years witness further increases in drug abuse [27].

Adolescents and youth constitute a significant percentage of the Iranian population [28]. The prevalence of drug abuse is low among Iranian students, but is increasing among younger ages (i.e., students in high school) [29-31]. Although previous studies have investigated drug abuse in Iran, no study is available on drug abuse behaviors, particularly among university freshmen. Furthermore, to the best of our knowledge, no study has examined the drug abuse behaviors of freshmen using latent class analysis (LCA). Thus, the present study aimed to investigate drug abuse behaviors among Iranian university freshmen using the LCA method.

## MATERIALS AND METHODS

This cross-sectional study was conducted between December 2013 and January 2014 in 4 cities in Iran: Tabriz (the capital of East Azerbaijan Province), Qazvin (the capital of Qazvin Province), Karaj (the capital of Alborz Province), and Khoramabad (the capital of Lorestan Province). Multi-stage random sampling was used to select 5,252 first-semester freshman students. Students were selected using a random proportional cluster sampling methodology that considered the type of university and major. The academic year starts on September 23 at Iranian universities. Therefore, students were selected approximately 3-4 months after they entered university. All selected students were invited to complete a survey questionnaire designed to collect data on socio-demographic characteristics and risk-taking behaviors. A total of 4,940 (94.1%) students completed the questionnaire and signed an informed consent form. Permission to conduct the study was obtained from the ethics committee of Tabriz University of Medical Sciences.

The survey questionnaire was tested on 132 freshman university students to examine its face validity and reliability (retest after 2 weeks). The pilot test input was used to modify the instrument/ questionnaire. A minimum Spearman’s correlation coefficient of 0.81 was obtained. To enhance the validity of students’ self-reports, they were assured of the strict confidentiality of their responses and the fact that they could not be recognized based on their responses. The participants also were informed about the voluntary nature of their participation in the study and their right to refuse or skip any questions.

To assess students’ substance abuse behaviors, the 4 indicator variables of cigarette smoking, hookah smoking, alcohol consumption, and drug abuse were used. Participants were classified by cigarette smoking status as never smokers, experimenters (having smoked fewer than 100 cigarettes in one’s lifetime regardless of current smoking status), and regular smokers (occasional users and regular smokers). Respondents were categorized by hookah smoking status as non-users, experimenters (those who had only tried hookah), occasional users, and regular hookah smokers (hookah use at least once per week). Participants were classified by alcohol consumption as never drinkers, experimenters, and those who had consumed alcohol at least once in the past month. Students were classified by drug abuse behavior as never and ever users (if the student had ever used any illicit drugs such as Ritalin, methamphetamine, ecstasy, cannabis, opium, or heroin).

### Statistical analysis

LCA was used for data analysis. To perform LCA, the 4 observable variables (i.e., indicators) of cigarette smoking, hookah smoking, alcohol use, and drug abuse were used to assess substance abuse behaviors among students as a latent variable. After finalizing the model, we entered age, sex, marital status, residence status, having a family member who smoked, and having a close friend who smoked as risk factors in the LCA model. The risk factors were included into a latent class model to identify characteristics that predicted membership in the various subgroups (latent classes). A likelihood ratio test was used for hypothesis testing in LCA with risk factors. All analyses were performed using proc-LCA in SAS version 9.2 (SAS Institute Inc., Cary, NC, USA).

## RESULTS

The participants’ age ranged from 17.0 to 34.0 years (mean, 20.6± 2.4 years). The majority of students in the sample were female (62.9%) and 6.2% were married. Only 24.8% of students had close friend(s) who were smokers, and 25.5% had a family member who smoked. Of the participants, 54.5% were living with their parents and the remaining 45.5% were living in a dormitory or shared accommodation. A summary of students’ substance abuse behaviors is presented in Table 1. The prevalence of all the substance abuse behaviors was generally low, although hookah smoking was more prevalent than other substance abuse behaviors.

On the basis of the 4 substance abuse indicators (cigarette smoking status with 3 levels, hookah smoking status with 4 levels, alcohol consumption with 3 levels, and drug abuse with 2 levels), there were 72 possible response patterns. We attempted to fit the LCA models with 1-6 classes. The G^2^ statistic test and its p-value, Akaike information criterion, and Bayesian information criterion were computed for each LCA model. With the exception of the 3-classes model, all models were statistically significant at the 0.05 level, indicating that the estimated models were not equivalent to the population model. Based on the model selection criteria and the interpretability of the results of the models, it was concluded that the model with 3 latent classes was most appropriate for the data. The results of the LCA model with 3 classes model showed that the differences between the expected and observed frequency of response patterns were not statistically significant (G^2^ =57.8; df=45; p= 0.10).

After finalizing the model, we entered age, sex, marital status, residence status, having a family member who smoked, and having a close friend who smoked as risk factors in the LCA model. Table 2 presents the prevalence of each latent class, item-response probabilities, and the odds ratios (ORs) of risk factors associated with latent class membership. The probability of membership in each latent class is shown in the first row of Table 3. Approximately 82.8, 16.1, and 2.1% of students were classified in the healthy, hookah experimenter, and unhealthy subgroups, respectively. The conditional probabilities of responses for each substance abuse behavior are listed in the middle rows in Table 3. These probabilities form the basis for the interpretation and labeling of the latent classes. Latent class 3, the unhealthy subgroup, was characterized by a high probability of responding with an advanced level for all the substance abuse behaviors. Individuals in this latent class were more likely to report that they had engaged in all the substance abuse behaviors investigated in this study. In contrast, those in latent class 1, the healthy subgroup, were more likely to report not engaging in any of the substance abuse behaviors. Latent class 2, the hookah experimenter subgroup, had a high probability of reporting hookah smoking, but not other substance abuse behaviors.

The ORs of membership in each class, compared to the first class, for the independent variables are also reported in Table 3. As can be seen in the Table 3, a 1-year increase in the age of the respondent increased the risk of membership in classes 2 or 3, compared to class 1. Similarly, being male increased the risk of membership in classes 2 or 3, compared to class 1. Marital status and living with one’s parents (in comparison to living in a dormitory or shared accommodation) did not change the risk of membership in classes 2 or 3. Moreover, having a family member or a close friend who smoked increased the risk of membership in classes 2 or 3.

## DISCUSSION

The results of this study indicated that the prevalence of cigarette smoking (experimenters and regular smokers), hookah smoking (occasional users and those who used hookah at least once per week), alcohol consumption (those who had consumed alcohol at least once in the past month), and drug abuse among university freshman students was 10.8, 17.0, 4.0, and 3.7%, respectively. These percentages are lower than have been reported for Iranian university students and high school students [18,29]. The higher prevalence of hookah smoking among university freshman students than the use of other substances may have been observed because hookah smoking in Iran generally starts during adolescence in an occasional manner before university, meaning that freshman university students are usually familiar with hookah smoking [32,33]. Moreover, hookah smoking is relatively common among all Iranian students, especially among university students [15,18,32,34,35].

According to the final fitted model for investigating drug abuse behaviors, approximately 82.8% of the freshmen belonged to the healthy subgroup, while 2.1% belonged to the unhealthy subgroup. In contrast, in a recently conducted study in Tabriz, 77.1 and 86.2% of male and female students, respectively, were classified into the healthy subgroup, while 13.3 and 4.3% of male and female students, respectively, were classified into the unhealthy subgroup [18]. The higher rate of drug abuse behaviors in that study among university students may have been due to various factors related to the conditions and environment of the universities. A study by Skidmore et al. [36] concluded that freshmen were exposed to a higher risk of drug abuse. In addition, admission to a university is recognized as a time of particular vulnerability to drug abuse [25]. Other studies have also shown that the onset of drug abuse among students was mainly in the first year of university education, although the following years were also associated with an increase in drug abuse [26,27].

The results of this study indicated that males and older age had statistically significantly positive relationships with membership in classes 2 and 3 of drug abuse behaviors, compared to class 1. These results are consistent with the findings of similar studies in Iran, which found that being male, being older, and having spent more time in the university environment were positively associated with drug abuse [18,32,37,38]. The relationship between male and drug use might be specific to the students and the drugs studied in this analysis. In other words, there might be no such relationship between sex and other types of drugs. For example, in some studies, the use of certain drugs, such as amphetamines, has been reported to be more prevalent among females [39].

A study by Mohammadpoorasl et al. [18] examined high-risk behaviors of Iranian students in 2011 and observed a positive and statistically significant relationship between being single and cigarette/hookah smoking among males. Similarly to a study by Sabahy et al. [32], the results of the present study showed no significant relationships between the marital status of students and drug abuse behaviors. In this study, single students exhibited fewer highrisk behaviors than the Iranian university students who participated in the study conducted by Mohammadpoorasl et al. [18]. As the current study focused on freshmen, while the study by Mohammadpoorasl et al. [18] focused on all university students, the lower rate of drug abuse (i.e., the tendency for students to report lower-ranked categories of drug abuse behavior) found in the present study may have been due to the shorter presence of freshman students in the university environment.

Various studies have demonstrated the role of parental support and discipline in reducing high-risk behaviors among young adults and students [37,40,41]. Family support is believed to be a major factor contributing to quitting drugs among students [42]. According to the findings of this study, although 45.5% of the freshman students were not living with their parents, no significant relationship was observed between living with one’s parents and drug abuse behavior. This might mean that living far from one’s parents and the lack of parental support among freshman students did not lead to higher drug abuse, whereas a longer stay in the university environment might change this relationship. According to Locke et al. [24], the sense of being apart from parents might be a reason associated with such changes in the environment and continuing to live in the new environment.

Based on the findings of this study, the presence of a smoker in the family was significantly related to membership in classes 2 and 3 of drug abuse behaviors, compared to class 1. Previous studies have reported that the presence of a drug abuser in the family was positively associated with drug abuse among other family members [37,39]. In this study, the highest OR was found for the relationship between having an intimate drug-abusing friend and belonging to classes 2 or 3. Previous studies have recognized the importance of this factor, which has a positive relationship with the onset of drug abuse, increased drug abuse, and other high-risk behaviors [32,38,43].

A major strength of the present study is its large and representative sample. However, this study also has some limitations. First, although a cross-sectional study design provides evidence of associations between variables, it provides no evidence of causality. Second, the study relied on self-reported data. Although we went to great lengths to ensure confidentiality and anonymity, we had no way of assessing the underreporting of substance abuse behaviors. Third, we did not have information on the behaviors of the participants during adolescence. Therefore, it is difficult to determine whether the risk behaviors analyzed in the present study emerged after participants entered university or were maintained from adolescence.

The overall prevalence of drug abuse behaviors among Iranian university freshmen was found to be low, and only approximately 2.1% of freshmen showed an unhealthy pattern of substance abuse behaviors. In addition, we found that older age, being male, and having a close friend or family member who was a smoker may serve as risk factors for substance abuse behaviors.

## Figures and Tables

**Table 1.** Prevalence of substance abuse behaviors among first-semester university freshmen in Iran

Variables	n (%)	95% CI	
		LL	UL
Cigarette smoking			
Never	4,385 (89.2)	88.3	90.0
Experimenter	248 (5.0)	4.5	5.7
Regular	283 (5.8)	5.1	6.4
Hookah smoking			
Never	3,239 (65.9)	64.6	67.3
Experimenter	836 (17.0)	16.0	18.1
Occasional user	675 (13.7)	12.8	14.7
At least once per week	162 (3.3)	2.8	3.8
Alcohol consumption			
Never	4,482 (90.9)	90.1	91.7
Experimenter	253 (5.1)	4.6	5.8
At least once in the past month	196 (4.0)	3.5	4.6
Drug abuse			
No	4,708 (96.3)	95.8	96.8
Yes	180 (3.7)	3.2	4.3

CI, confidence interval; LL, lower limit; UL, upper limit.

**Table 2.** Comparison of LCA models with different latent classes according to the model selection statistics

Latent statuses (n)	Parameters estimated (n)	G^2^	df	p-value	Maximum log-likelihood	AIC	BIC
1	8	1,946.6	63	<0.001	-9,353.8	1,962.6	1,976.1
2	17	156.3	54	<0.001	-8,378.7	190.3	219.0
3	26	57.8	45	0.10	-8,330.9	109.8	153.7
4	35	51.4	36	0.05	-8,319.7	121.4	180.6
5	44	48.8	27	0.006	-8,301.9	136.8	211.2
6	53	37.3	18	0.005	-8,294.2	143.3	232.9

LCA, latent class analysis; AIC, Akaike information criterion; BIC, Bayesian information criterion.

**Table 3.** The model of substance abuse behaviors with 3 latent classes and the corresponding risk factors among first-semester university freshmen in Iran

Variables	Latent classes			p-value
	Healthy	Hookah experimenter	Un-healthy	
Prevalence (%)	82.8	16.1	2.1	
Indicators in each latent class				
Cigarette smoking				
Never	0.982^1^	0.550^1^	0.000	
Experimenter	0.012	0.243	0.101	
Regular	0.006	0.208	0.899^1^	
Hookah smoking				
Never	0.788^1^	0.093	0.010	
Experimenter	0.148	0.293	0.108	
Occasional user	0.065	0.450^1^	0.573^1^	
At least once per week	0.000	0.164	0.309	
Alcohol consumption				
Never	0.988^1^	0.627^1^	0.007	
Experimenter	0.009	0.242	0.255	
At least once in the past month	0.004	0.132	0.739^1^	
Drug abuse				
No	0.990^1^	0.890^1^	0.459	
Yes	0.010	0.110	0.541^1^	
Covariates (odds ratio)^2^				
Age				
Crude	1.00	1.49	1.32	<0.001
Adjusted	1.00	1.13	1.19	<0.001
Male (ref: female)				
Crude	1.00	3.78	12.33	<0.001
Adjusted	1.00	1.59	1.88	0.002
Married (ref: single)				
Crude	1.00	0.85	1.91	0.26
Adjusted	1.00	1.02	0.96	0.41
Living with parents				
Crude	1.00	0.67	0.81	0.005
Adjusted	1.00	1.06	1.03	0.51
Having a family member who smoked				
Crude	1.00	2.99	4.77	<0.001
Adjusted	1.00	3.68	6.33	<0.001
Having a close friend who smoked				
Crude	1.00	16.41	69.18	<0.001
Adjusted	1.00	9.10	48.49	<0.001

1 Larger item-response probabilities to facilitate interpretation.

2 Crude model: only 1 risk factor; Adjusted model: all risk factors.
